# Psychophysiological correlates of professional experience among female teachers of secondary schools in Kazakhstan

**DOI:** 10.1371/journal.pone.0355232

**Published:** 2026-08-21

**Authors:** Nurlan Smagulov, Olzhas Zhamantayev, Ayaulym Arystanbay, Dmitry Ageev, Gulzhazira Turlybekova, Aizhan Aitkulova

**Affiliations:** 1 Faculty of Biology and Geography, Karaganda Buketov University, Karaganda, Kazakhstan; 2 School of Public Health, Karaganda Medical University, Karaganda, Kazakhstan; 3 Kokshetau Higher Medical College, Kokshetau, Kazakhstan; Ondokuz Mayıs Üniversitesi: Ondokuz Mayis Universitesi, TÜRKIYE

## Abstract

Teaching is widely documented as one of the most psychologically and physiologically demanding knowledge-intensive occupations, with chronic exposure to combined intellectual, emotional, and regulatory workloads placing teachers at elevated risk for autonomic dysregulation, cardiovascular strain, cognitive decline, and occupational burnout. A question that occupational health research has not adequately resolved is whether the functional consequences of career-long teaching are driven primarily by biological ageing or by the accumulation of occupationally specific stressor exposure, two processes that are conceptually distinct yet rarely examined separately. We assessed psychophysiological, emotional, and cognitive indicators in 103 female secondary school teachers in Karaganda, Kazakhstan, stratified by age and length of service. The assessment battery included heart rate variability recording, systolic and diastolic blood pressure measurement, the Ruffier physical performance index, the Well-being, Activity, and Mood questionnaire, the Spielberger-Khanin State Anxiety Inventory, the Landolt ring correction test, a digital selective attention test, and three sensorimotor reaction time measures. To allow direct comparison of the explanatory power and predictor composition of the two variables, separate multiple linear regression models were constructed with chronological age and length of professional service as dependent variables, with the full psychophysiological battery serving as independent variables in both. Chronological age and length of service were strongly intercorrelated (r = 0.915; p < 0.001), and partial correlation analysis showed that neither variable independently predicted digital test performance once the other was controlled. The age-as-dependent model was statistically significant and explained 42.2% of the variance (R^2^ = 0.422; adjusted R^2^ = 0.392; F = 14.166; p < 0.001), with significant predictors confined to physiological and cognitive domains: perceived activity (β = 0.417), digital test response time (β = 0.415), subjective well-being (β = −0.365), systolic blood pressure (β = 0.190), and correction test execution time (β = 0.171). The service-length model also showed strong explanatory coverage (R^2^ = 0.510; adjusted R^2^ = 0.479; F = 16.657; p < 0.001) and additionally captured the Ruffier index (β = −0.209), reactive anxiety (β = −0.189), and digital test response time (β = 0.345) as significant predictors alongside activity, well-being, and systolic blood pressure. These differences in predictor composition suggest that length of professional service is associated with a broader functional profile than chronological age, additionally capturing physical performance capacity and emotional reactivity. Taken together, the differential predictor structures indicate that biological ageing and cumulative occupational exposure operate through partially distinct functional pathways, and that treating the two variables as analytically interchangeable may obscure information relevant to the design of targeted occupational health monitoring programmes. These findings support the analytical differentiation of age and service length in occupational health research and provide a basis for developing career-stage-stratified preventive strategies for teachers.

## Introduction

Teaching occupies a distinctive position among knowledge-intensive professions, combining sustained intellectual effort with continuous emotional engagement, high communicative demands, and pronounced responsibility for outcomes that extend well beyond the classroom. Unlike many cognitive occupations in which stress exposure is episodic, teaching imposes a chronic and compounded workload in which lesson preparation, pupil assessment, parental communication, administrative reporting, and real-time conflict mediation occur simultaneously and without meaningful recovery intervals [1,2]. These conditions place teachers among the most consistently stress-exposed occupational groups documented across educational systems on multiple continents [3,4].

The physiological consequences of prolonged occupational stress in this population are well established. Chronic exposure to high-demand, low-recovery working conditions triggers dysregulation of the hypothalamic-pituitary-adrenal axis and the sympathetic branch of the autonomic nervous system, producing sustained elevations in cortisol, epinephrine, and norepinephrine [5]. Over time, these neuroendocrine shifts translate into measurable changes in cardiovascular function, including elevated resting heart rate, increased systolic and diastolic blood pressure, and reduced heart rate variability (HRV), as well as deterioration in cognitive performance, attention regulation, and sensorimotor reactivity [6,7]. These changes are not confined to subjective reports of fatigue or dissatisfaction, they manifest as objectively quantifiable shifts in psychophysiological indicators that can be assessed under standardized conditions. Burnout, defined as the triad of emotional exhaustion, depersonalisation, and reduced personal accomplishment [8], is a recognised downstream outcome of this chronic strain, with prospective studies confirming that it independently predicts incident hypertension, sleep disorders, and metabolic dysfunction in education workers [5,9]. The OECD Teaching and Learning International Survey estimated that between 25% and 40% of teachers across participating systems describe their occupational stress as high or very high [10], while UNESCO documented emotional exhaustion in 30–45% of teachers in middle-income countries [11]. The World Health Organization’s global framework on workers’ mental health identifies chronic occupational stress as a leading contributor to reduced work ability and premature labor-force exit among professional groups [12]. Together, these reports situate teaching at the intersection of occupational health, public health, and educational policy. It is a domain where psychophysiological research can yield both scientific insight and actionable preventive guidance. What has received considerably less attention is the question of attribution: whether the physiological and cognitive changes observed across a teaching career primarily reflect biological ageing that would occur regardless of occupation, or whether they are substantially shaped by the cumulative demands specific to professional practice.What has received considerably less attention is the question of attribution: whether the physiological and cognitive changes observed across a teaching career primarily reflect biological ageing that would occur regardless of occupation, or whether they are substantially shaped by the cumulative demands specific to professional practice.

In Kazakhstan, teaching is formally classified as high-intensity mental work characterised by sensory overload, emotional stressors, prolonged static posture, and physical inactivity [13]. The country’s general secondary education system employs approximately 341,000 teachers, of whom over 80% are women [14]. Kazakhstani teachers face structural stressors that amplify internationally documented risks: successive national educational reform cycles demanding rapid pedagogical adaptation, heavy administrative reporting obligations, elevated student-to-teacher ratios in urban schools, and limited access to institutional psychological support [10,12]. These conditions make the Kazakhstani teaching workforce a relevant and practically important population for examining the psychophysiological correlates of cumulative professional exposure.

Against this established evidence base, a specific and practically important gap remains. Studies of occupational health in teachers routinely include both chronological age and length of professional service as variables of interest, yet the two are almost invariably treated as interchangeable proxies for career progression, or one is controlled for when the other is examined, without investigating whether they capture distinct aspects of teachers’ functional state [15,16]. This analytical conflation is consequential. Chronological age primarily reflects biological maturation and senescence, including progressive changes in autonomic flexibility, cardiovascular reactivity, cognitive processing speed, and neuroendocrine regulation that occur as a function of biological time, largely independent of occupational context [17,18]. Length of professional service, by contrast, indexes cumulative exposure to occupationally specific stressors and the adaptive or maladaptive physiological adjustments that consolidate over years of practice [19,20]. These are not equivalent constructs. If they are associated with different psychophysiological profiles, treating them interchangeably may obscure which functional domains are primarily driven by biological ageing and which reflect the specific burden of sustained occupational exposure, with direct implications for how occupational health monitoring and prevention programmes are designed and targeted. The Job Demands-Resources model and Ilmarinen’s work-ability framework together provide the theoretical basis for this distinction. Within the JD-R model, when job demands chronically exceed available personal and organisational resources, emotional exhaustion and reduced regulatory capacity accrue through a depletion process driven primarily by exposure duration rather than biological age per se [19,21]. Demerouti and colleagues demonstrated that job demands are the primary antecedents of the exhaustion component of burnout, while resource deficits drive disengagement [22], a pattern that would be expected to generate service-length-specific associations in the psychophysiological indicators most sensitive to emotional regulation. Ilmarinen’s framework further supports the analytical separation of the two variables, concluding that professional ageing is multidimensional and encompasses biological, cognitive, and socio-occupational components that cannot be reduced to chronological age alone [17]. Both frameworks thus predict that biological ageing and cumulative occupational exposure should generate different functional signatures: age-driven changes concentrated in physiological and processing-speed domains, and service-length-driven changes extending into emotional regulation and physical fitness. Despite this conceptual foundation, the high collinearity between age and service length in typical working samples has led most investigators to examine them jointly rather than as analytically distinguishable constructs [15,16], leaving the prediction of divergent functional profiles largely untested in the empirical literature.

Three specific hypotheses guided the present investigation. First, length of professional service was predicted to be associated with a greater proportion of explained variance than chronological age in regression models incorporating a comprehensive battery of psychophysiological, emotional, and cognitive indicators, on the grounds that it more directly indexes cumulative occupationally specific stressor exposure. Second, the two variables were expected to differ in their predictor compositions: age was anticipated to show stronger associations with physiological indicators consistent with biological ageing, while length of service was expected to show stronger associations with emotional and regulatory indicators reflecting occupational adaptation. Third, given the near-certain high collinearity between the two variables in this sample, it was recognised from the outset that simple bivariate associations would be insufficient to resolve their independent contributions. Therefore we employed partial correlation analysis as an explicit diagnostic step prior to regression modelling, and the regression coefficients are interpreted as describing conditional associations rather than independent effects. The aim of the study was to compare the associations of chronological age and length of professional service with the psychophysiological, emotional, and cognitive functional state of female secondary school teachers in Kazakhstan, and to identify the functional domains most closely associated with each variable as a basis for developing targeted preventive strategies.

## Methods

### Study design

This investigation employed a cross-sectional observational design to assess the psychophysiological, emotional, and cognitive characteristics of secondary school teachers and to quantify their associations with chronological age and length of professional service. The primary analytical objective was comparative: to determine which of the two variables was associated with a broader and more explanatorily informative profile of teachers’ functional state, and to examine whether the two variables differed in the composition of their predictor structures. The cross-sectional design establishes statistical associations only.

### Study setting and participants

The study was conducted among teachers employed in secondary schools in Karaganda, the second largest city in Kazakhstan and the administrative center of Karaganda region. Data collection took place between February 3 to April 18, 2025. All examinations were performed in the morning, between 09:00 and 11:00, under standardized conditions (ambient temperature 22 ± 2°C, relative humidity 50–60%), in a dedicated quiet room within each school. Participants were instructed to refrain from caffeine consumption, smoking, and vigorous physical activity for at least two hours prior to assessment.

**Fig 1:**
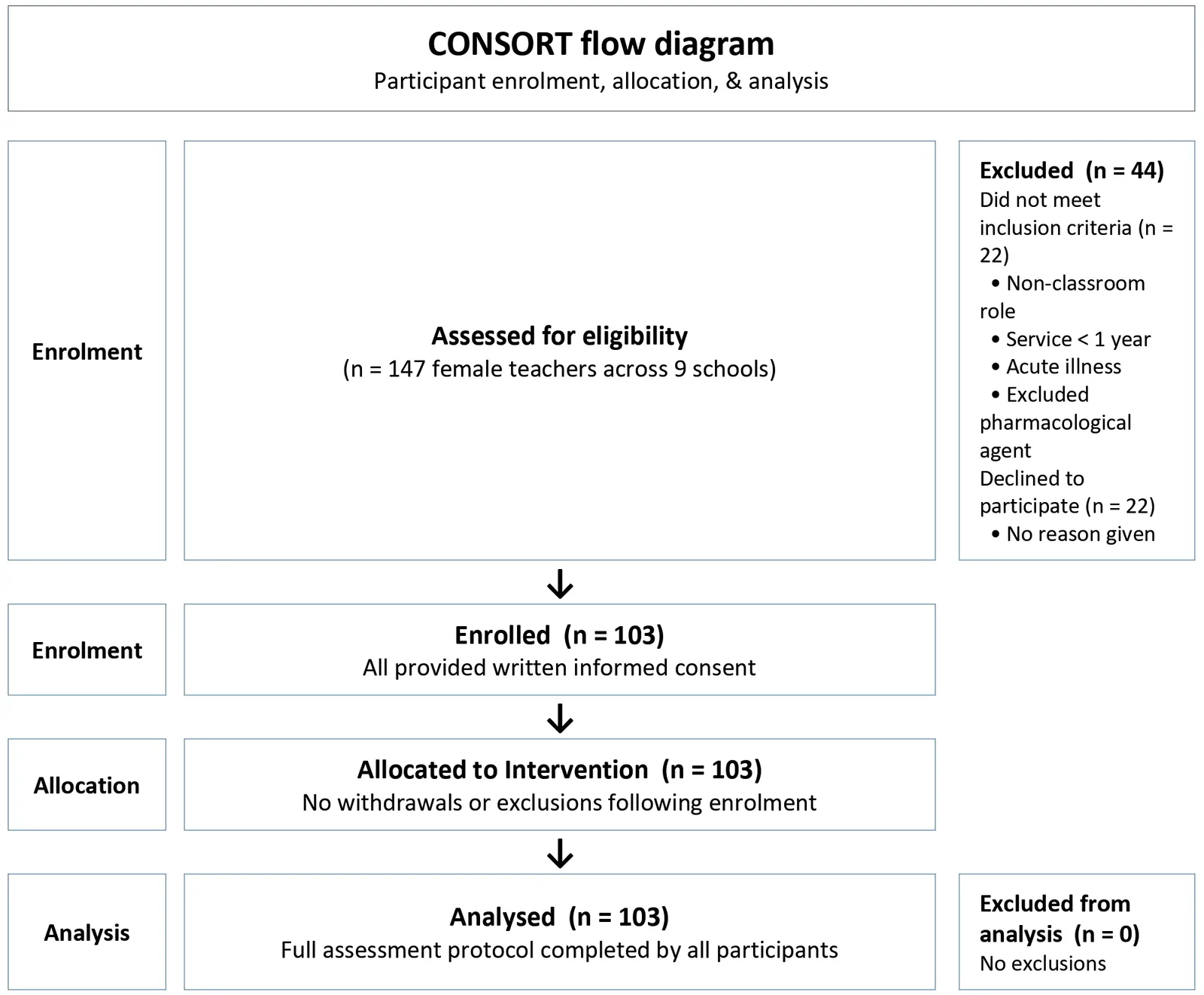
Participant recruitment flow.

A total of 147 female teachers across the nine participating schools were assessed for initial eligibility. Of these, 44 were excluded at the screening stage. Screening records identified 22 participants who did not meet one or more inclusion criteria (most commonly, employment in a non-classroom role, length of service shorter than one year, acute illness, or use of a pharmacological agent listed among the exclusion criteria). There were 22 people who declined to participate without providing a specific reason. The remaining 103 teachers provided written informed consent and were enrolled. No participants withdrew or were excluded following enrolment, and all 103 completed the full assessment protocol. Fig 1 presents the participant recruitment flow. Participants were eligible for inclusion if they were: (1) female; (2) employed as classroom teachers in a secondary school in Karaganda; (3) had accumulated at least one year of professional service; and (4) reported normal subjective well-being on the day of examination. Participants were excluded if any of the following applied: (1) acute infectious illness, exacerbation of a chronic condition, or hypertensive crisis on the day of examination; (2) cardiovascular, neurological, or endocrine disorders in a stage of decompensation; (3) use of pharmacological agents known to substantially alter heart rate or psychophysiological reactivity, including beta-blockers, antidepressants, and anxiolytics; (4) employment in a non-classroom capacity; (5) length of service shorter than one year; or (6) refusal to provide written informed consent.The sample was female by design. Male teachers in Kazakhstan predominantly occupy administrative posts or teach subjects such as physical education and basic military training, functions that differ substantially in their psychoemotional and cognitive demands from standard classroom instruction. All participants were actively employed in a classroom teaching role with a minimum of one year of professional service, representing subject specialisations across humanities, natural sciences, and technical disciplines. All participants worked a standard five-day schedule with a contractual teaching load of 16–18 academic hours per week.To facilitate intergroup comparisons, the sample was stratified by both age and length of service. By age, participants were allocated to three subgroups: younger than 30 years (n = 35; M = 25.97 ± 0.44 [SE]; SD = 2.60), 30–45 years (n = 54; M = 35.63 ± 0.59 [SE]; SD = 4.36), and older than 45 years (n = 14; M = 47.43 ± 0.43 [SE]; SD = 1.60). By length of professional service, participants were allocated to three subgroups: up to 10 years (n = 41; M = 3.41 ± 0.43 [SE]; SD = 2.75), 10–20 years (n = 46; M = 12.74 ± 0.36 [SE]; SD = 2.46), and more than 20 years (n = 16; M = 25.31 ± 0.75 [SE]; SD = 3.00). The cut-off values reflect career-stage transitions that are functionally meaningful within the Kazakhstani educational context and align with groupings used in the international literature [10, 20]. The smaller sizes of the oldest age subgroup (n = 14) and the longest-service subgroup (n = 16) are acknowledged as limitations that may affect the stability of subgroup-level comparisons.

### Ethical considerations

The study was approved by the Local Bioethics Commission of Karaganda Medical University (Protocol No. 17, dated October 22, 2024) and was conducted with formal authorization from the Karaganda City Department of Education. All procedures were carried out in full accordance with the principles of the Declaration of Helsinki (revised 2013). Written informed consent was obtained from each participant prior to enrollment. Participation was entirely voluntary.

### Assessment instruments

HRV was selected as the primary indicator of autonomic regulatory function, consistent with its designation as the international standard for non-invasive autonomic assessment [6]. A resting electrocardiogram was recorded in the seated position for five minutes using the Varikard 2.4 hardware and software complex (Neurosoft, Russia; sampling frequency 1,000 Hz). Time-domain parameters extracted included: heart rate (HR, bpm), standard deviation of normal-to-normal intervals (SDNN, ms), mode amplitude (AMo, %), variation range (VR, s), and coefficient of variation (CV, %). Integral indices included the stress index of regulatory systems (SI) and the centralisation index (CI) [23].

Systolic blood pressure (SBP) and diastolic blood pressure (DBP) were measured using a validated automated sphygmomanometer after a five-minute rest, with three sequential measurements at two-minute intervals and the mean value used for analysis. Physical performance was assessed using the Ruffier index, a standardised functional stress test quantifying cardiovascular recovery efficiency following controlled physical exertion [24,25]. Current subjective functional state was assessed using the Well-being, Activity, and Mood (WAM) questionnaire [26]. Situational emotional stress was assessed using the State subscale of the Spielberger-Khanin State-Trait Anxiety Inventory (STAI), referred to as reactive anxiety [27]. Attentional stability and visual information processing speed were assessed using the Landolt ring correction test, a standardised paper-based proofreading paradigm in which participants scan rows of optotypes (Landolt rings with gaps oriented in different directions) and cancel specified targets within a fixed time period; outcome variables include execution time (seconds), number of rings reviewed, total information processed (bits), information processing speed (bits/s), and error count [28]. Selective attention and psychomotor speed were evaluated using a computerised digit-based selective attention test (referred to throughout as the digital test), in which participants respond to target digits displayed on screen among non-target distractors; mean response time (seconds) was the primary outcome variable. Three reflexometric tests assessed the latent period of sensorimotor reaction: the auditory motor reaction (AMR) test, visual motor reaction (VMR) test, and complex visual motor reaction (CoVMR) test [29]. Variables with substantially skewed distributions (IPS, SDNN, SI, AMR, VMR, CoVMR) were log-transformed prior to analysis and are designated with the prefix ‘ln.’

### Statistical analysis

All statistical analyses were performed using IBM SPSS Statistics version 26.0 (IBM Corp., Armonk, NY, USA). Data are presented as mean ± standard deviation (M ± SD) for the full sample and as M ± SE for subgroup descriptions unless otherwise specified. Normality was evaluated using the Shapiro-Wilk test supplemented by skewness and kurtosis coefficients. Robustness of parametric analyses was confirmed by comparing Pearson and Spearman correlations across all key variable pairs; differences did not exceed 0.02 to 0.06 for any pair.

Given the high collinearity between age and service length, an analytical strategy incorporating both simple and partial correlations was adopted prior to regression modelling. Two separate multiple linear regression models were then constructed: one with chronological age as the dependent variable and one with length of professional service as the dependent variable, with the full set of psychophysiological, emotional, and cognitive indicators as independent variables in both. Because this design is uncommon, its rationale warrants explicit statement: the goal was to identify which psychophysiological indicators are most closely associated with each career variable in a multivariate context, and whether the two variables differ in the breadth and composition of their associated functional profiles. No multiple comparison correction was applied across the correlation analyses; this is acknowledged as a limitation, and all p-values should be interpreted with appropriate caution. Multicollinearity was evaluated using tolerance (threshold: > 0.5) and the variance inflation factor (VIF; threshold: < 2.0). Residual independence was assessed using the Durbin-Watson statistic. Model fit was evaluated using R^2^, adjusted R^2^, and the overall F-statistic. Standardised regression coefficients (β) are reported alongside unstandardised coefficients (B), standard errors, and 95% confidence intervals (CIs). All significance tests were two-tailed, and the threshold for statistical significance was set at p < 0.05.

## Results

### Descriptive statistics and distributional characteristics

Descriptive statistics for all psychophysiological, emotional, and cognitive indicators are presented in Table 1. Mean SBP and DBP values (105.68 ± 10.94 and 68.54 ± 8.21 mmHg, respectively) fell within normotensive ranges, and mean heart rate was 75.90 ± 8.18 bpm. The Ruffier index averaged 5.37 ± 1.49, indicating satisfactory physical performance. Subjective well-being and activity scores (5.66 ± 0.77 and 5.05 ± 0.99, respectively) were within the mid-range of the WAM scale. Reactive anxiety averaged 30.88 ± 7.01 points, corresponding to a low-to-moderate level. Correction test execution time averaged 46.98 ± 10.83 seconds and digital test mean response time was 5.03 ± 1.34 seconds.

**Table 1:** Descriptive statistics for psychophysiological, cognitive, and emotional indicators in the study sample (n = 103).

Variable	Mean	SD	Skewness	Kurtosis
Reactive anxiety	30.88	7.01	0.742	−0.229
Well-being	5.66	0.77	−0.835	0.315
Activity	5.05	0.99	−0.462	−0.767
Mood	5.99	0.74	−0.734	0.210
Correction test: execution time (s)	46.98	10.83	0.160	0.271
Correction test: rings reviewed	24.81	5.62	−0.594	0.078
Processed information, Q (bits)	169.80	40.70	−0.246	−0.466
Processed information per character, q	0.85	0.20	−0.246	−0.463
Errors, N	8.86	5.45	0.591	−0.250
lnIPS (bits/s, log-transformed)	1.28	0.29	−0.476	1.700
SBP (mmHg)	105.68	10.94	0.207	0.460
DBP (mmHg)	68.54	8.21	0.169	−0.174
Ruffier index	5.37	1.49	−0.268	0.590
HR (bpm)	75.90	8.18	0.328	1.425
lnSDNN (ms, log-transformed)	3.50	0.48	−0.760	2.413
AMo (%)	54.07	15.84	0.647	0.737
Variation range (s)	0.17	0.07	0.621	−0.247
CV (%)	25.49	9.93	0.824	1.104
lnSI (log-transformed)	5.24	0.57	−0.471	−0.565
CI	0.18	0.08	0.467	0.053
lnAMR (ms)	5.56	0.31	0.422	−0.614
lnVMR (ms)	5.64	0.32	0.671	−0.559
lnCoVMR (ms)	6.17	0.19	0.302	−0.914
Digital test (s)	5.03	1.34	0.550	−0.613

Note. SD = standard deviation; SBP = systolic blood pressure; DBP = diastolic blood pressure; HR = heart rate; AMo = mode amplitude; CV = coefficient of variation; SI = stress index; CI = centralization index; AMR = auditory motor reaction; VMR = visual motor reaction; CoVMR = complex visual motor reaction; IPS = information processing speed. Variables prefixed with “ln” were log-transformed prior to analysis.

### Simple and partial correlations with cognitive indicators

Chronological age and length of professional service were very strongly intercorrelated (r = 0.915; p < 0.001), confirming the high collinearity that motivated the two-stage analytical strategy. Simple Pearson correlations showed that digital test response time was significantly associated with both chronological age (r = 0.476; p < 0.001) and length of professional service (r = 0.471; p < 0.001), indicating that slower selective attention performance was associated with increasing biological age and accumulating professional experience in roughly equal measure (Table 2). Partial correlation analysis substantially qualified this picture. When length of professional service was held constant, the association between chronological age and digital test performance became statistically non-significant (partial r = 0.125; p = 0.208). Conversely, when chronological age was held constant, the association between service length and the digital test also disappeared (partial r = 0.102; p = 0.307). These corrected values are reported in Table 2. The mutual attenuation of both associations when the other variable is controlled is mathematically expected given the near-perfect collinearity (r = 0.915); neither variable independently accounts for variance in selective attention performance once the shared variance with the other is removed. This finding does not imply functional interchangeability across all indicators, as the regression analyses below demonstrate.

**Table 2:** Simple and partial correlations of chronological age and length of professional service with digital test performance (n = 103).

Variable pair	Correlation type	r	p	Controlled variable
Age – digital test	Simple (Pearson)	0.476	< 0.001	None
Service length – digital test	Simple (Pearson)	0.471	< 0.001	None
Age – digital test	Partial	0.125	0.208	Length of service
Service length – digital test	Partial	0.102	0.307	Age

Note. r = Pearson correlation coefficient; p = two-tailed significance level. Highlighted rows contain corrected values.

## Regression models: chronological age and length of professional service

The multiple linear regression model with chronological age as the dependent variable was statistically significant (F = 14.166; p < 0.001) and explained 42.2% of the variance in age (R^2^ = 0.422; adjusted R^2^ = 0.392). Five predictors contributed independently and significantly to the model (Table 3). Perceived activity level emerged as the strongest positive predictor (β = 0.417; p < 0.001), followed closely by digital test response time (β = 0.415; p < 0.001), indicating that older teachers on average had slower selective attention while simultaneously reporting higher perceived activity. Well-being was a significant negative predictor (β = −0.365; p < 0.001), reflecting a pattern in which higher subjective well-being scores were associated with younger age. SBP contributed independently as a positive predictor (β = 0.190; p = 0.021), consistent with the expected age-related trend of rising resting SBP. Correction test execution time also reached significance as a positive predictor (β = 0.171; p = 0.035), indicating slower attentional processing in older teachers. The model was characterized by the predominance of physiological and cognitive predictors, with emotional and autonomic regulatory indicators not achieving significance within this model structure.

**Table 3:** Multiple linear regression model results for chronological age and length of professional service as dependent variables (n = 103).

Dependent variable	Predictor	B	SE	β	t	p	95% CI	VIF
Age	Constant	5.932	7.931	—	0.748	0.456	[−9.81, 21.67]	—
	Digital test	2.418	0.466	0.415	5.195	< 0.001	[1.50, 3.34]	1.072
	Correction test execution time	0.123	0.058	0.171	2.140	0.035	[0.01, 0.24]	1.073
	Activity	3.286	0.748	0.417	4.392	< 0.001	[1.80, 4.77]	1.514
	Well-being	−3.678	0.958	−0.365	−3.840	< 0.001	[−5.58, −1.78]	1.513
	SBP	0.135	0.057	0.190	2.353	0.021	[0.02, 0.25]	1.091
Length of service	Constant	−7.855	9.557	—	−0.822	0.413	[−26.83, 11.12]	—
	Digital test	2.056	0.450	0.345	4.565	< 0.001	[1.16, 2.95]	1.118
	SBP	0.247	0.055	0.339	4.493	< 0.001	[0.14, 0.36]	1.117
	Activity	3.772	0.724	0.468	5.208	< 0.001	[2.33, 5.21]	1.582
	Well-being	−4.253	0.983	−0.412	−4.328	< 0.001	[−6.20, −2.30]	1.775
	Ruffier index	−1.119	0.404	−0.209	−2.773	0.007	[−1.92, −0.32]	1.117
	Reactive anxiety	−0.215	0.103	−0.189	−2.082	0.040	[−0.42, −0.01]	1.607

Note. B = unstandardised regression coefficient; SE = standard error; β = standardised regression coefficient; CI = 95% confidence interval; VIF = variance inflation factor; SBP = systolic blood pressure. Age model: R^2^ = 0.422; adjusted R^2^ = 0.392; F = 14.166; p < 0.001. Service-length model: R^2^ = 0.510; adjusted R^2^ = 0.479; F = 16.657; p < 0.001.

The regression model with length of professional service as the dependent variable was statistically significant and explained 51.0% of the variance (R^2^ = 0.510; adjusted R^2^ = 0.479; F = 16.657; p < 0.001). Six predictors contributed significantly, spanning a wider functional range than the age model (Table 3). In the subjective functional state domain, perceived activity was again the strongest positive predictor (β = 0.468; p < 0.001), replicating and strengthening the activity-age association, while well-being was a strong negative predictor (β = −0.412; p < 0.001). In the cardiovascular domain, SBP emerged as a more prominent positive predictor than in the age model (β = 0.339; p < 0.001), and digital test response time contributed positively (β = 0.345; p < 0.001). Two predictors appeared exclusively in this model: the Ruffier index as a negative predictor (β = −0.209; p = 0.007) and reactive anxiety as a negative predictor (β = −0.189; p = 0.040). Because higher Ruffier index values indicate poorer cardiovascular recovery efficiency, the negative coefficient means that longer service was associated with lower, and thus more favourable, Ruffier index values.

### Model diagnostics and comparative fit

Both models were statistically significant at p < 0.001 by ANOVA. Collinearity diagnostics confirmed the absence of problematic multicollinearity in both models. VIF values ranged from 1.07 to 1.51 in the age model and from 1.12 to 1.78 in the length of service model, with all values remaining well below the threshold of 2.0. Corresponding tolerance values ranged from 0.66 to 0.93 and from 0.56 to 0.89 respectively, both comfortably exceeding the minimum acceptable value of 0.5. Durbin-Watson statistics were 2.018 for the age model and 1.756 for the length of service model, both within the acceptable range and indicating the absence of residual autocorrelation. Standardised residuals in the age model ranged from −2.31 to +2.07 and in the service-length model from −1.85 to +2.43, with no values exceeding the ± 3 SD criterion. A comparative summary of model fit statistics is provided in Table 4.

**Table 4:** Comparative fit statistics for the age and length of professional service regression models (n = 103).

Fit index	Age model	Length of service model
Multiple correlation coefficient (R)	0.650	0.714
Coefficient of determination (R²)	0.422	0.510
Adjusted R²	0.392	0.479
F statistic (ANOVA)	14.166 (p < 0.001)	16.657 (p < 0.001)
Durbin-Watson statistic	2.018	1.756
VIF range	1.07 to 1.51	1.12 to 1.78
Tolerance range	0.66 to 0.93	0.56 to 0.89
Standardized residual range	−2.31 to +2.07	−1.85 to +2.43
Number of significant predictors	5	6

**Fig 2:**
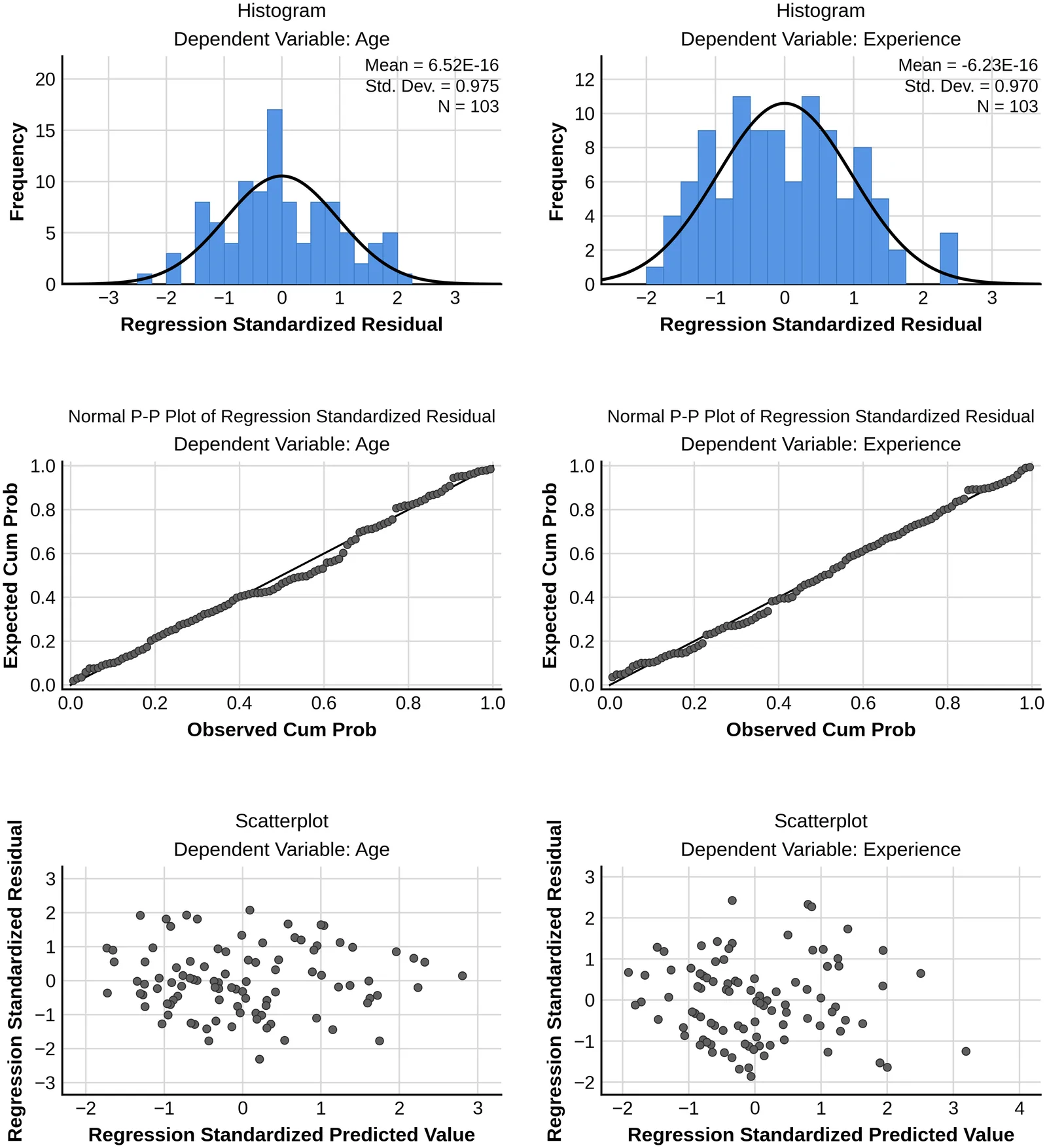
Regression model diagnostics for the age and length of professional service models. Panels display from top to bottom: histograms of standardized residuals, normal probability-probability plots, and scatterplots of standardized residuals against standardized predicted values. Left column: age model. Right column: length of professional service model.

Graphical residual diagnostics for both models are presented in Fig 2. Histograms of standardized residuals illustrated distributions closely approximating normality without pronounced skew or asymmetry. Normal probability-probability plots showed close correspondence between observed and theoretically expected cumulative probabilities, confirming the adequacy of the normality assumption for residuals.

## Discussion

The central finding of this study is that length of professional service was associated with a broader and more functionally diverse predictor profile than chronological age among female secondary school teachers in Kazakhstan. The service-length regression model accounted for 51.0% of the variance compared with 42.2% for the age model, and its predictor structure spanned cardiovascular, physical fitness, emotional, and cognitive domains simultaneously, a profile that the age model, characterised by predominantly physiological and cognitive predictors, did not replicate. These differences are consistent with the theoretical position that cumulative occupational exposure constitutes a functionally distinct pathway from biological ageing [17,19], one that progressively taxes emotional regulatory reserves and physical fitness capacity in ways that cannot be attributed to biological time alone. A cross-cutting feature of both models deserves particular note: perceived activity emerged as the single strongest positive predictor in each, yet it co-occurred with declining well-being, a dissociation that the subsections below interpret as probable evidence of compensatory engagement. These are statistical associations observed in a cross-sectional sample, and causal inferences cannot be drawn.

The association between lower subjective well-being and older age in the present sample is broadly consistent with work-ability research in teachers. Hlaďo and colleagues documented that older age and burnout were both independently associated with lower work ability among Czech upper-secondary school teachers [30], a pattern directionally similar to the well-being decrement observed here, although the outcome measures differ. Skaalviks found that teachers’ subjective functional state was substantially moderated by school goal structure and perceived self-efficacy, suggesting that organisational conditions amplify or attenuate the age-related patterns documented in psychophysiological studies [31]. The finding that a single composite assessment battery could meaningfully differentiate between the two career variables is itself noteworthy. Scoping reviews of teacher health consistently identify years of teaching experience, workload, and class size as key correlates of burnout and psychological strain, yet few studies have attempted to examine whether these variables are distinguishable from chronological ageing at the psychophysiological level [32,33]. The present results suggest they are, at least in the domains of physical fitness, emotional reactivity, and cardiovascular function.

### Occupational stress and cardiovascular burden: systolic blood pressure associations

The association between longer service duration and higher SBP is consistent with prospective evidence linking cumulative occupational stress to sustained cardiovascular reactivity and progressive resting blood pressure elevation [34]. The plausible mechanisms are well characterised: chronic activation of the sympathetic-adrenal-medullary axis in response to sustained psychosocial demands may contribute, over years of exposure, to increased peripheral vascular resistance and elevated resting SBP [35]. Among teachers specifically, the emotionally demanding and time-pressured occupational environment has been associated with heightened cardiovascular reactivity in multiple studies [5,36]. That SBP appeared as a significant predictor in both models is consistent with the hypothesis that occupationally related stressor accumulation may amplify age-related cardiovascular risk [5]. These interpretations remain speculative given the cross-sectional design. A meta-analysis and systematic review by Liu and colleagues, covering eleven studies encompassing 5,696 participants, found that chronic psychosocial stress was associated with a more than twofold increase in hypertension risk (OR = 2.40; 95% CI: 1.65 to 3.49), with sustained occupational stress identified as a particularly consistent predictor [37]. A study reviewed the mechanisms linking job strain to blood pressure elevation, noting that the persistent sympathetic activation associated with low job control and high job demands produces not only acute cardiovascular reactivity but, over time, structural vascular changes consistent with early hypertension [38]. Experimental and clinical literature on stress-related hypertension links chronic emotional stress to increased sympathetic activity, with possible involvement of neuroglial and oxidative mechanisms [39].

The observation that SBP carried a larger standardised coefficient in the service-length model (β = 0.339) than in the age model (β = 0.190) provides limited but suggestive evidence that the blood pressure associations in this sample are more closely linked to occupational exposure than to biological ageing per se. A study, examining sympathetic nervous system responses to acute psychosocial stress in male physicians with clinical burnout, found that burnout was associated with a blunted epinephrine response to the Trier Social Stress Test, interpreted as indicating sympathoadrenal medullary dysfunction consistent with the allostatic load framework [40]. Although the direction of autonomic dysregulation in burnout is context-dependent and varies by burnout stage and severity, the convergent finding across occupational groups is that prolonged stress exposure produces measurable shifts in cardiovascular regulatory function that are not fully explained by chronological age [39,40]. It should be noted that the present sample’s mean SBP of 105.68 mmHg falls within the normotensive range, indicating that the associations described here operate within adaptive limits rather than in a clinically hypertensive context.

### Physical performance capacity and sedentary occupational profile

The inclusion of the Ruffier index as a significant negative predictor in the service-length model, but not the age model, suggests that prolonged professional exposure may be accompanied by a decline in physical fitness reserves not fully accounted for by biological ageing within the age range of this sample. This is consistent with evidence that sedentary occupational profiles, a defining characteristic of teaching given the prolonged static posture and physical inactivity inherent in classroom instruction, are associated with progressive compromise of cardiorespiratory fitness [12,41]. The practical implication is that physical deconditioning in teachers may be attributable at least partly to occupational conditions rather than biological ageing alone. Physical activity outside the workplace was not measured, however, limiting interpretation of this finding. The evidence base on sedentary occupations and physical performance is directly relevant here. A systematic review and meta-analysis by Prince and colleagues, covering 132 studies with data from 15,619 participants, found that office workers accumulated the greatest sedentary time and among the lowest step counts per day relative to other occupational groups, with clear associations between objectively measured sedentary time and adverse cardiometabolic profiles [42]. Teaching, while not classified as office work in the conventional sense, shares the defining features of low occupational physical activity: extended static standing or seated instruction, minimal locomotion during the working day, and insufficient recovery periods between cognitively demanding tasks. Ekblom-Bak and colleagues examined the physical activity paradox in the context of workers with low cardiorespiratory fitness, finding that only 25% to 50% of Swedish workers in occupations with higher aerobic demands had sufficient fitness to sustain good health throughout their employment [43]. While teachers do not perform high aerobic demand work, the broader point, that occupationally enforced inactivity progressively erodes the physical reserves available to cope with workload demands, applies directly to this population.

Kenny and colleagues reviewed the physiological adaptations to ageing in the context of occupational physical capacity, noting an average 20% decline in physical work capacity between the ages of 40 and 60 years due to reductions in aerobic and musculoskeletal capacity [44]. Critically, their review concluded that differences in habitual physical activity substantially influence the variability in this decline, and that well-organised worksite health interventions encouraging physical activity during working hours could delay age-related injury and illness. The present finding that the Ruffier index was associated with service length rather than with chronological age per se, within the relatively young sample (mean age 33.95 years), suggests that occupational factors may accelerate physical deconditioning beyond the trajectory expected from biological ageing alone in this population. This interpretation requires longitudinal confirmation.

### Autonomic regulation and heart rate variability

HRV parameters did not emerge as significant regression predictors in either model in the present study. HRV indices in the present sample were within adaptive ranges, suggesting that the sample as a whole had not reached a state of severe autonomic dysregulation, consistent with evidence that HRV suppression tends to be most pronounced under extremely sustained or severe emotional demands [6]. This finding does not imply that autonomic regulation was unaffected by professional exposure; it may instead reflect the sensitivity thresholds of short-term resting HRV recording in a non-clinical sample. A systematic review of HRV and occupational stress by Järvelin-Pasanen and colleagues, covering ten peer-reviewed studies published between 2005 and 2017, confirmed that heightened occupational stress is consistently associated with reduced HRV, particularly with decreases in RMSSD and high-frequency spectral power indicating attenuated parasympathetic activation [45]. Lo and colleagues investigated HRV in 120 workers across burnout categories and found a positive association between HRV and job seniority, suggesting that more experienced workers may demonstrate partial autonomic adaptation [46], a finding consistent with the habituation hypothesis discussed below. Kanthak and colleagues, in a population-based sample of 410 participants from the Dresden Burnout Study, found that emotional exhaustion specifically, rather than other burnout dimensions, was negatively associated with vagally-mediated HRV (RMSSD) during both a stressor and resting conditions, even after adjusting for established autonomic modulators including age and body mass index [47]. Taken together, these findings suggest that the absence of significant HRV predictors in the present regression models may partly reflect the early-to-moderate career stage of the sample rather than insensitivity of HRV to occupational stressors more broadly.

A study specifically examining gender differences in autonomic and psychological stress responses among educators found that, despite reporting higher levels of stress and anxiety, female teachers demonstrated higher HRV than male counterparts, suggesting a stronger parasympathetic regulatory response in women [48]. This gender-specific finding is directly relevant to the all-female composition of the present sample, as it indicates that the autonomic protective capacity of female teachers may partially buffer the expression of HRV suppression within a normative range even under conditions of elevated occupational stress, providing a possible additional explanation for the non-significance of HRV parameters in the current regression models.

### Emotional functioning, burnout, and professional experience

The appearance of reactive anxiety as a significant predictor exclusively in the service-length model provides evidence that emotional reactivity is more closely associated with cumulative occupational exposure than with biological ageing per se, within the age range represented in this sample. This is consistent with the JD-R framework, which proposes that sustained exposure to high job demands progressively depletes personal regulatory resources, compromising emotional resilience over time [19]. The theoretical underpinnings of the JD-R model have been substantially developed over the past two decades. Demerouti and colleagues originally demonstrated that job demands are primarily related to the exhaustion component of burnout, while the absence of job resources is primarily related to disengagement [22]. Bakker and colleagues subsequently extended this framework by integrating self-regulation perspectives, proposing that short-term job strain translates into enduring burnout through consistently high job demands combined with failed self-regulation, a process in which maladaptive coping strategies such as cognitive inflexibility and self-undermining behaviour become increasingly probable as strain accumulates [49]. In the context of teaching, this dynamic is particularly relevant as background theory: as emotional demands accumulate over a career, teachers with limited access to institutional support and autonomy may become progressively less able to deploy adaptive regulatory strategies. However, the negative direction of the reactive anxiety coefficient in the present data (β = −0.189) indicates that more experienced teachers showed lower, not higher, situational anxiety in the regression framework.

A scoping review by Agyapong and colleagues, covering burnout, stress, anxiety, and depression among teachers worldwide, identified years of teaching, class size, and job satisfaction as consistent correlates of burnout across multiple educational systems [32]. The review found that burnout prevalence ranged from 25.12% to 74% across included studies when moderate to severe levels were considered, underscoring the scale of the occupational health burden in this population. Importantly, the review identified that years of teaching contributed to burnout independently of age in several included studies, consistent with the differential predictor profiles observed in the present investigation. Pakdee and colleagues, in a cross-sectional study of 410 teachers in Thailand, found that working hours and work experience, but not age alone, significantly affected the depersonalisation dimension of burnout, while age and workload together predicted emotional exhaustion [50]. A study of 38 teachers in the Khazar district using the Maslach Burnout Inventory found that teaching experience was directly proportional to the degree of emotional burnout, with all respondents reaching the third degree of emotional burnout having more than 20 years of professional experience [51].

The well-being subscale of the WAM questionnaire contributed as a significant negative predictor in both regression models, with a slightly larger standardised coefficient in the service-length model (β = −0.412) than in the age model (β = −0.365). This pattern suggests that subjective well-being deterioration is a cross-cutting correlate of both biological ageing and professional exposure in this sample, though more closely associated with career progression than with biological age. Nwoko et al. identified personal capabilities, socioemotional competence, professional relationships, and organisational conditions as determinants of teachers’ occupational well-being, while career stage may influence how well-being is experienced and reported [33]. Their review specifically noted that career stage moderated the expression of wellbeing decline, consistent with the present finding that well-being was more strongly associated with service length than with age alone.

Springer and colleagues, in a cross-sectional study of Polish academic teachers using the JD-R and transactional stress models, found a strong relationship between an excessively demanding work environment and both occupational burnout and chronic fatigue, with health-promoting behaviours including sleep and vacation use only slightly moderating this relationship [52]. This finding is consistent with the present observation that activity and well-being, the WAM subscales most directly reflecting motivational engagement and perceived health, were among the strongest predictors in both regression models, suggesting that subjective functional state indicators capture the cumulative strain of professional exposure more sensitively than objective physiological measures within the normative range.

### Cognitive performance and attentional functioning

Digital test response time and correction test execution time indicate that attentional processing speed declined with both increasing age and accumulating service duration, consistent with well-established trajectories of cognitive change in healthy working populations [53]. The standardised coefficient was somewhat larger in the age model (β = 0.415) than in the service-length model (β = 0.345), indicating that attentional slowing was more closely associated with chronological age than with service length in a direct coefficient comparison, consistent with cognitive ageing as a primary contributor to slower selective attention processing. Auditory motor reaction time did not contribute independently to either model after accounting for the other predictors, and sensorimotor processing speed is therefore not identified as a differentiating characteristic of service length in this dataset.

A narrative review by Giorgi and colleagues on cognitive impairment in the ageing working population concluded that chronic occupational stress is a recognised contributor to cognitive decline through mechanisms involving a chronic decrease in the inhibitory processes of neurological pathways, noting that organisational strategies for managing stress in elderly workers with the aim of preventing cognitive impairment remain scarce [54]. Golonka and colleagues, in a study of cognitive impairments specifically attributable to occupational burnout, demonstrated that error processing and indices of reactive and proactive cognitive control were measurably impaired in workers meeting burnout criteria relative to non-burned-out controls, supporting the inference that cognitive slowing in experienced workers may reflect both burnout-related neural resource depletion and age-associated processing speed decline [55]. The observation that older teachers reported higher perceived activity alongside lower well-being remains noteworthy. One plausible interpretation draws on compensatory effort theory: workers experiencing declining physiological and cognitive efficiency may intensify behavioural engagement to maintain acceptable performance standards, a strategy that is simultaneously effortful and accompanied by diminished subjective wellbeing [56]. This pattern is consistent with Ilmarinen’s work-ability model, in which the gap between work demands and individual functional resources widens progressively with biological age [17]. An alternative interpretation could be that the positive association between activity and both age and service length reflects an occupational socialisation effect, whereby experienced teachers develop a higher baseline standard of professional engagement that is perceived as active effort regardless of underlying physiological state.

### Reactive anxiety: Habituation versus healthy worker survivor effect

The negative direction of the reactive anxiety association with service length requires careful interpretation and deserves explicit caution. On one reading, it may reflect adaptive habituation: teachers with longer careers may develop greater psychological resilience and lower situational reactivity through repeated exposure to occupational stressors, a process consistent with the concept of stress inoculation [57]. Evidence for this habituation hypothesis exists in the broader occupational stress literature: Schaufeli documented that experienced professionals in high-demand occupations sometimes report lower state anxiety than early-career counterparts [21]. An equally plausible explanation is a healthy-worker or survivor effect: teachers with high baseline anxiety and low stress tolerance may disproportionately exit the profession before reaching longer service durations, leaving a selected sample of emotionally resilient individuals in the higher-experience subgroups.

The healthy worker survivor effect (HWSE) is a well-documented source of bias in occupational epidemiology, arising from the concomitance of three associations: the relationship between job status and subsequent exposure, the relationship between job status and disease, and the relationship between prior exposure and job status [58]. In practical terms, workers who develop health problems in response to occupational exposure are more likely to leave the workforce, such that surviving long-tenure cohorts are systematically healthier than the population from which they were drawn. Brown and colleagues reviewed the methodological implications of the HWSE, noting that its presence leads to underestimation of harmful occupational exposures in cross-sectional and cohort studies comparing workers of different service durations [58]. Massamba and colleagues provided direct evidence for the HWSE in the context of psychosocial work-related factors and hypertension: in a prospective cohort study of 9,188 white-collar workers, they found that retirement, by eliminating ongoing job strain exposure, was associated with reductions in hypertension prevalence of up to 39% in older women, and demonstrated that the HWSE was present across multiple gender and age subgroups [59].

In the present study, the negative association between reactive anxiety and service length is statistically significant but modest (β = −0.189). Both mechanisms, genuine stress habituation and differential attrition of anxiety-prone teachers, would produce the same directional result in a cross-sectional sample, and current data cannot distinguish between them. Future longitudinal studies tracking anxiety trajectories within intact teacher cohorts, ideally with records of reasons for professional exit, would be needed to resolve this ambiguity. The practical implication follows directly from the interpretive uncertainty: an absence of elevated reactive anxiety among experienced teachers should not be treated as evidence of occupational emotional resilience or as grounds for deprioritising emotional health support in this group. Occupational health programmes should therefore apply anxiety screening regardless of service length, rather than using experienced teachers’ apparently lower anxiety scores as a basis for reducing monitoring intensity.

### Relevance to the Kazakhstani context and middle-income country settings

Several features of the Kazakhstani educational context plausibly contribute to the pattern of associations observed. Teaching loads at 16–18 academic hours per week plus substantial extracurricular and administrative obligations are comparable to those in high-stress educational systems documented in the international literature. The gender composition of the sample reflects a structural feature of the educational workforce in Kazakhstan with direct relevance to occupational health interpretation. The all-female composition of the sample should be interpreted in the context of Kazakhstan’s gendered division of unpaid work. Evidence from Kazakhstan indicates that women’s labour-market participation coexists with persistent gender asymmetry in housework and childcare, which may shape work-life strain among female teachers [60]. The psychophysiological consequences of this dual burden, including its contribution to autonomic imbalance associated with chronic psychosocial stress, are likely to interact with the occupationally determined associations identified in the present data, though the cross-sectional design and the absence of measures of domestic workload prevent formal evaluation of this interaction.

Kazakhstan’s educational context adds structural conditions that may intensify internationally documented teacher stressors, including repeated reform cycles, administrative reporting demands, and limited access to systematic occupational health monitoring for teaching staff. This interpretation should be framed cautiously, since organisational workload, institutional support, and caregiving burden were not directly measured in the present study. At the broader system level, psychosocial occupational health has been identified as an increasingly relevant area for middle-income countries, where work-stress research is expanding but translation into prevention practice remains uneven [61]. For teachers specifically, LMIC-focused literature points to the need for better evidence on psychosocial interventions adapted to school settings and local implementation conditions [62]. Consistent with WHO and ILO guidance, occupational health programmes should include regular assessment of psychosocial risks and organisational interventions that reduce work-related sources of mental strain [63,64]. The present findings support the development of teacher monitoring systems in Kazakhstan that stratify risk by length of professional service and combine brief psychophysiological assessment with preventive psychological support.

### Practical and preventive implications

The regression predictor structures identified in this study have direct implications for the design of occupational health monitoring and prevention programs for teachers. The indicators that distinguished the length of service model from the age model, specifically reactive anxiety, and the Ruffier index represent functional domains that are plausibly amenable to targeted intervention independent of age-related biological change. Interventions addressing situational anxiety, physical fitness maintenance, and central nervous system recovery through structured rest and workload redistribution are supported by the existing evidence base and are practically implementable within school settings [31,57].

The cardiovascular indicators, particularly systolic blood pressure elevation with increasing service duration, highlight the importance of routine blood pressure screening as a component of occupational health protocols for teachers, especially those with more than 10 years of service. The present data provide an empirical basis for advocating the incorporation of brief, standardized psychophysiological assessments, including HRV recording, systolic blood pressure measurement, Ruffier testing, and validated measures of emotional state and reactive anxiety, into routine occupational health examinations for teaching staff, stratified by service length rather than age cohort alone.

The consistent and sizeable contributions of the Well-being and Activity subscales of the WAM instrument to both regression models support the use of brief, validated subjective functional state screening as a practical monitoring tool within school-based occupational health programs, particularly in resource-limited settings where more resource-intensive assessments may not be routinely feasible. The WAM instrument requires minimal equipment and administration time, making it suitable for periodic monitoring, and its consistent performance as a predictor in both models supports its validity as a functional health marker in this population [26].

## Limitations

Several limitations should be considered when interpreting these findings. The cross-sectional study design establishes statistical associations only, it is not possible to determine from these data whether the psychophysiological profiles observed in more experienced teachers reflect the cumulative effects of professional exposure, differential selection and retention processes, or both.

The sample was drawn from a single city, limiting generalisability. The female-only composition accurately reflects the gender structure of the Kazakhstani teaching workforce but prevents evaluation of sex-based differences. Participation rates were not formally recorded across all schools, meaning that selection bias cannot be formally evaluated. Teachers are nested within nine schools, and standard OLS regression does not account for potential within-school clustering, therefore multilevel modelling would provide a more rigorous analytical framework in future studies with larger samples.

The regression models did not incorporate several organisational variables identified by the OECD and UNESCO as significant contributors to occupational stress, including class size, institutional leadership quality, collegial support, and perceived autonomy [10]. Physical activity outside the workplace, sleep quality, and caregiving burden were also not assessed, representing potential sources of residual confounding. No correction for multiple comparisons was applied across the correlation analyses, and all p-values should be interpreted conservatively. The small sizes of the oldest age subgroup (n = 14) and the longest-service subgroup (n = 16) may affect the stability of subgroup-level regression coefficients, and independent replication in larger multi-site samples is warranted.

## Conclusions

In this cross-sectional sample of female secondary school teachers in Kazakhstan, length of professional service was associated with a broader and more functionally diverse predictor profile than chronological age. While age was associated primarily with physiological and cognitive indicators consistent with biological maturation, service length additionally and independently captured associations with physical performance capacity and emotional reactivity. The service-length model accounted for 51.0% of the variance compared with 42.2% for the age model, with six and five significant predictors respectively. The differential predictor structures support the position that biological ageing and cumulative occupational exposure operate through partially distinct functional pathways. Treating the two variables as analytically interchangeable, as most occupational health studies implicitly do, may suppress signals in the physical fitness and emotional regulation domains that are most directly responsive to occupationally specific stressor accumulation and therefore most amenable to targeted workplace intervention. These conclusions are specific to the present cross-sectional sample; longitudinal replication in larger, multi-site cohorts is necessary before they can be generalised or translated into formal programmatic recommendations.

## Supporting information

S1 File DB PONE-D-26-12868 anonymized. (XLSX)
